# Editorial: Plant Viruses, Volume II: Molecular Plant Virus Epidemiology and Its Management

**DOI:** 10.3389/fmicb.2021.756807

**Published:** 2021-10-25

**Authors:** Rajarshi Kumar Gaur, Akhtar Ali, Xiaofei Cheng, Kristiina Mäkinen, Bright Agindotan, Xifeng Wang

**Affiliations:** ^1^Department of Biotechnology, Deen Dayal Upadhyaya Gorakhpur University, Gorakhpur, India; ^2^Department of Biological Science, The University of Tulsa, Tulsa, OK, United States; ^3^Key Laboratory of Germplasm Enhancement, Physiology and Ecology of Food Crops in Cold Region of Chinese Education Ministry, College of Agriculture, Northeast Agricultural University, Harbin, China; ^4^Department of Microbiology, Viikki Plant Science Center, University of Helsinki, Helsinki, Finland; ^5^Science & Technology (S&T) Beltsville Laboratory, USDA APHIS, Beltsville, MD, United States; ^6^State Key Laboratory for Biology of Plant Diseases and Insect Pests, Institute of Plant Protection, Chinese Academy of Agricultural Sciences, Beijing, China

**Keywords:** plant virus, high throughput sequencing, transmission, mixed infections, virus-host plant interactions, resistance, CRISPR/Cas

The International Committee on Taxonomy of Viruses (ICTV) lists nearly 2,000 different species of plant viruses. Plant viruses are recognized as economically important plant pathogens, because they cause significant losses in a variety of crops that we rely on. Due to the global transportation of agricultural products and planting materials and intensive agricultural farming practices, emerging new viruses and reemerging known viruses significantly threat food security. There is currently no efficient or long-term control measures for many of the plant viruses that cause serious diseases. On these lines, Zhang Z. et al. describes the occurrence and distribution of orthotospoviral diseases and their possible management strategies in China.

A big advancement in ecological plant virus research has been achieved by high throughput sequencing (HTS). HTS allows quick identification of most, if not all, viruses in plants without a need for prior information, which viruses might be present. In their expert review, Maclot et al. highlights the many opportunities that careful use of the HTS method opens up in understanding the behavior of plant virus populations in cultivated and wild plants. Velasco and Padilla consider the possibility to use HTS in certification schemes based on the results of a comparison between HTS of small RNAs and other diagnostic methods in grapevine plants. The prospects of HTS based diagnosis in controlling plant virus diseases are bright.

Plant viruses enter the plant cell by mechanical injury, when transported by insects or nematodes feeding on them or via parasitic agents such as fungi. Virus-insect vector interaction studies are essential for understanding the connections between plant viral transmission routes and virus epidemics. In their review, Zhang L. et al., discuss fijivirus outbreaks, which occur in connection to migrations of the planthopper vectors. The authors give insights into several aspects of vector transmission including the effects of viruses on the behavior and physiology of vector insects. An interesting finding presented by Du et al., is that the RNA-silencing suppressor NSs of tomato spotted wilt virus reduces the monoterpene volatiles in a way that increases the attractiveness of the plant for the transmission vector thrip *Frankliniella occidentalis*. In their paper, McGreal et al. studied the possibility to use ground cover plants as an alternative controlling strategy to reduce transmission of grapevine leafroll-associated virus 3 by mealybugs. Zhang J. et al., asked the question about the role of virus-induced expression of circular RNAs in the vector *Laodelphax striatellus* on rice black-streaked dwarf virus infection. Chen et al., found that the virus concentrations in the salivary gland of the leafhopper vector *Nephotettix cincticeps* were higher during the intermittent period than during the transmitting period of rice dwarf virus, a plant reovirus, which led the authors to discuss a threshold-regulated release of viruses from the insect vectors.

Plant responses to mixed infections vary, but because of their many possible adverse consequences, it is important to understand how viruses interact in co-infected susceptible plants. A previously undescribed interaction of a potyvirus watermelon mosaic virus protein P1 with a crinivirus cucurbit yellow stunting disorder virus protein P25 was proposed to contribute to the complex response of the host to this virus pair (Domingo-Calap et al.). The authors demonstrate reduced RNA silencing suppression activity of the crinivirus P25 in this interaction. An interaction that reduced the activity of RNA silencing suppressor of another crinivirus cucurbit chlorotic yellows virus was discovered by Yang et al., In this case the interactor was a ribosomal protein that could negatively affect virus accumulation. These studies suggest that the silencing suppressor proteins interact with various proteins and many antiviral defense pathways during crinivirus infections. It also simply is amazing in how many ways geminiviral AC2 silencing suppressor proteins interfere with post-transcriptional gene silencing, transcriptional gene silencing and other plant defense pathways as can be learned from the review article by Veluthambi and Sunitha.

Basal immunity of non-host plant species protects plants against most plant virus diseases. Also host plants may express basal resistance at certain developmental stages. The factors underlying the mechanism of age-related resistance against a geminivirus, tomato leaf curl virus, were studied by against a geminvirus tomato yellow leaf curl virus were studied by Zhang J.-R. et al. They suggest that salicylic acid plays a major role and raise the possibility to deploy the cues obtained from these studies in managing the disease. Knowledge on the molecular mechanisms of plant virus- host interactions provides ideas and novel targets for resistance breeding. The mechanism of resistance toward rice stripe virus, achieved by expressing coat protein encoding transcripts, relates to RNA silencing in Arabidopsis as demonstrated in Sun et al. While RNA silencing-mediated resistance provides one option, it is important to explore other possibilities as well. Identification of host factors that either promote or suppress infections by interacting with viral proteins is an important study field in the search of possible novel resistance targets. Increasing body of evidence indicate that specific nuclear factors are involved in systemic spread of several viruses. The study by Alazem et al., reveals that the control of systemic spread of bamboo mosaic virus is connected to a correct homeostasis of a nuclear high mobility group protein and to processes it regulates. As reviewed in Kozieł et al., the components of cell wall affect the spread of the virus and the apoplast- and symplast-based defense mechanisms both in susceptible and resistant plants. Engineering antiviral mechanisms to host plants to prevent and control plant viruses has been and will continue to be an effective management strategy. Modern DNA/RNA editing tools based on clustered regularly interspaced short palindromic repeats (CRISPR) and CRISPR-associated Cas nucleases are reviewed by Cao et al. They discuss the challenges and opportunities provided by CRISPR/Cas systems for controlling different plant viruses by targeting either viral sequences or host susceptibility genes.

Finally, we would like to show our gratitude to all the authors who contributed to the Research Topic. The articles cover a broad spectrum of plant host-virus interactions and management strategies and open several channels for study by providing major insights into these complex relationships.

## Author Contributions

All authors listed have made a substantial, direct and intellectual contribution to the work, and approved it for publication.

## Conflict of Interest

The authors declare that the research was conducted in the absence of any commercial or financial relationships that could be construed as a potential conflict of interest.

## Publisher's Note

All claims expressed in this article are solely those of the authors and do not necessarily represent those of their affiliated organizations, or those of the publisher, the editors and the reviewers. Any product that may be evaluated in this article, or claim that may be made by its manufacturer, is not guaranteed or endorsed by the publisher.

